# RAS and beyond: the many faces of the neurofibromatosis type 1 protein

**DOI:** 10.1242/dmm.049362

**Published:** 2022-02-21

**Authors:** Corina Anastasaki, Paola Orozco, David H. Gutmann

**Affiliations:** Department of Neurology, Washington University School of Medicine, St Louis, MO 63110, USA

**Keywords:** Neurofibromin, RAS, Cyclic AMP, Tumor suppressor

## Abstract

Neurofibromatosis type 1 is a rare neurogenetic syndrome, characterized by pigmentary abnormalities, learning and social deficits, and a predisposition for benign and malignant tumor formation caused by germline mutations in the *NF1* gene. With the cloning of the *NF1* gene and the recognition that the encoded protein, neurofibromin, largely functions as a negative regulator of RAS activity, attention has mainly focused on RAS and canonical RAS effector pathway signaling relevant to disease pathogenesis and treatment. However, as neurofibromin is a large cytoplasmic protein the RAS regulatory domain of which occupies only 10% of its entire coding sequence, both canonical and non-canonical RAS pathway modulation, as well as the existence of potential non-RAS functions, are becoming apparent. In this Special article, we discuss our current understanding of neurofibromin function.

## Abstract (precis)

**Summary:** Neurofibromatosis type 1 has provided unprecedented opportunities to define the importance of RAS regulation in health and disease. Emerging research has also unveiled the role of non-canonical RAS and RAS-independent signaling in this RASopathy.

## Introduction

Neurofibromatosis type 1 (NF1) is an autosomal-dominant condition, affecting 1 in 3000 individuals worldwide (Evans et al., 2010; Uusitalo et al., 2015). Although all people with NF1 are born with a germline mutation in the *NF1* gene, thousands of different germline mutations in this gene have been reported (Koczkowska et al., 2018). Moreover, the spectrum of clinical manifestations in any given individual is highly variable, even within families. Children and adults are at risk for developing numerous medical and neuropsychiatric problems, including hyperpigmented spots on their skin (café-au-lait macules, axillary and inguinal freckling) and iris (Lisch nodules), learning deficits, autism symptomatology, seizures, vision loss, depression, chronic pain, cardiac malformations, bone defects, neuroendocrine tumors, brain and nerve tumors, and breast cancer (Ferner and Gutmann, 2013; Gutmann et al., 2017). This extreme degree of clinical heterogeneity represents a major barrier to risk assessment and proactive medical management.

In the 1980s, a concerted and coordinated effort was made to collect families with NF1 in order to identify the causative gene. This included the formation of the National Neurofibromatosis Foundation (NNFF) and the establishment of uniform consensus clinical criteria to enable an accurate diagnosis of NF1. In 1990, two groups, one headed by Dr Francis S. Collins (University of Michigan) and another by Dr Ray White (University of Utah), announced the cloning of the *NF1* gene on chromosome 17q11.2 using converging methods and numerous patient DNA samples. The discovery of the *NF1* gene confirmed the genetic basis for this condition, and helped to establish that NF1 results from germline mutations in this gene.

**Fig. 1.**
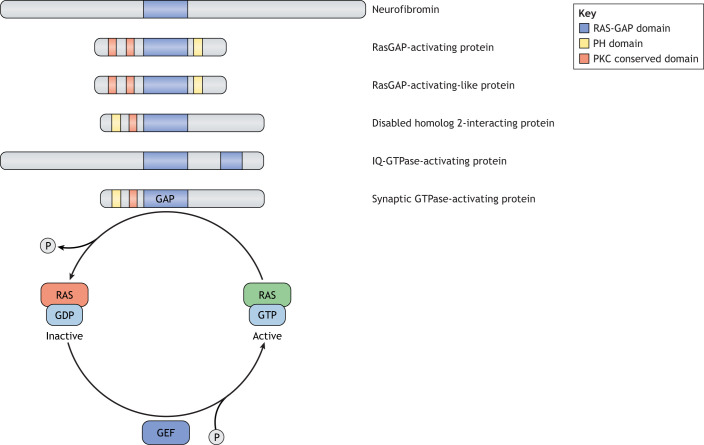
**Neurofibromin is a member of the RAS-GAP superfamily of proteins.** Schematic representation of neurofibromin and other RAS-GAPs, highlighting the conserved protein domains and the activity of the RAS-GAP domain. RAS-GAPs catalyze GTP to GDP hydrolysis by stimulating the intrinsic GTPase activity of RAS, leading to RAS inactivation. Conversely, GEFs promote the exchange of GDP for GTP, converting RAS to its active GTP-bound conformation. GAP, GTPase-activating protein; GDP, guanosine-5′-diphosphate; GEF, guanine exchange factor; GTP, guanosine-5′-triphosphate; P, phosphate; PH, plekstrin homology; PKC, protein kinase C.

Further sequence analysis revealed that the *NF1* gene encoded a large protein of 2818 amino acids, termed neurofibromin, with partial sequence similarity to a family of proteins known to function as negative regulators of the RAS proto-oncogene. These negative regulators are termed RAS GTPase-activating proteins (RAS-GAPs) and all share a small 300-amino acid RAS-binding domain (Fig. 1). RAS proteins are membrane-tethered molecules that are activated by guanine exchange factors (GEFs) in response to growth factor receptor (e.g. receptor tyrosine kinases) or G-protein-coupled receptor (GPCR) ligand binding. GEFs, in turn, promote the exchange of GDP for GTP on the RAS proteins, converting RAS from its inactive GDP-bound form to its active GTP-bound conformation. Conversely, this activation is negatively controlled by gate-keeping GTPase-activating proteins (GAPs), which facilitate the hydrolysis of the GTP moiety by stimulating the intrinsic GTPase activity of RAS, leading to RAS inactivation.

Exons 27-34 of the *NF1* gene encode the 300-amino acid structural and functional RAS-GAP homology domain that alone is sufficient to accelerate RAS-GTP hydrolysis and result in decreased RAS activity (Ballester et al., 1990, 1989). In this manner, neurofibromin functions as a GAP to negatively regulate the activity of all three classic RAS proteins (HRAS, NRAS, KRAS) (Ballester et al., 1990; Martin et al., 1990; Xu et al., 1990), but may also regulate other RAS-regulated proteins, like RRAS and MRAS (Huang et al., 2004; Rey et al., 1994; Weber et al., 2021). In individuals with NF1, loss-of-function *NF1* mutations decrease neurofibromin GAP function, resulting in increased RAS activity (Basu et al., 1992; DeClue et al., 1992), and, in turn, drive cell hyperproliferation. Notably, overexpression of the neurofibromin GAP domain is sufficient to normalize the increased RAS activity and cell hyperproliferation in *Nf1*-deficient mouse cells and tissues (Hiatt et al., 2001; Ismat et al., 2006). Taken together, these findings support the idea that many clinical features of NF1 are caused by impaired neurofibromin RAS proto-oncogene regulation.

**Fig. 2.**
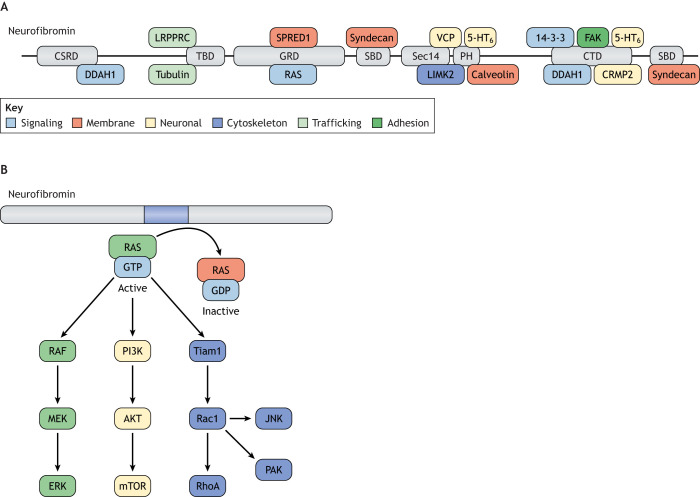
**Neurofibromin binding partners and canonical RAS signaling.** (A) Neurofibromin is a large molecule, containing numerous predicted protein domains, which have been shown to interact with a diverse set of binding partners. Protein domains are as follows: cysteine-serine rich domain (CSRD), GAP-related domain (GRD), C-terminal domain (CTD), pleckstrin homology domain (PH), syndecan-binding domain (SBD), Sec-14 domain (Sec14), tubulin-binding domain (TBD). The position of domains with which the interacting proteins bind is shown. Neurofibromin binding partners are categorized by function in the key. Adapted from Ratner and Miller (2015). This image is not published under the terms of the CC-BY license of this article. For permission to reuse, please see Ratner and Miller (2015). (B) The neurofibromin GRD functions to accelerate the conversion of active GTP-bound RAS to its inactive GDP-bound form. Active RAS can activate numerous distinct downstream effectors to result in increased RAF/MEK.

The assignment of neurofibromin as a RAS-GAP shifted the focus of NF1 research on RAS downstream signaling, and the development of therapeutic agents inhibiting RAS or RAS effector activity and function. However, the functionally characterized RAS regulatory domain only comprises 10% of the neurofibromin molecule (Scheffzek et al., 1998), while the function of the rest of the protein remains largely unknown. Given the diverse number of clinical manifestations involving many distinct cell types (https://www.proteinatlas.org/ENSG00000196712-NF1/tissue), as well as the growing number of potential neurofibromin-interacting proteins (Fig. 2A), which will likely expand following the recent crystallization of the neurofibromin dimer protein (Lupton et al., 2021; Naschberger et al., 2021), it is conceivable that neurofibromin may have functions beyond simple canonical RAS pathway regulation. In this Special article, we will detail what is known about *NF1* protein function and discuss evidence supporting both RAS-dependent and RAS-independent mechanisms for neurofibromin regulation of cell biology.

## Neurofibromin and canonical RAS-dependent signaling

### RAS-dependent signaling in NF1-associated cancer

Owing to the critical role of RAS in cell cycle regulation and tissue growth, the majority of studies investigating neurofibromin function have focused on its RAS-GAP properties, especially in the context of cancer, where oncogenic mutations affecting RAS signaling drive tumorigenesis. Although sporadic *NF1* mutations are frequently observed in human cancers (Cancer Genome Atlas Research Network, 2008; Way et al., 2018), individuals with NF1 are born with one mutated (non-functional) copy of the *NF1* gene (germline *NF1* gene mutation). While heterozygosity for a germline *NF1* mutation has biological consequences, such as learning and attention deficits, and autism (Bajenaru et al., 2001; Costa et al., 2002, 2001; Cui et al., 2008; Li et al., 2005; Maloney et al., 2018; Molosh et al., 2014; Molosh and Shekhar, 2018; Omrani et al., 2015; Silva et al., 1997), a single *NF1* mutation is not sufficient for tumor formation. Loss of *NF1* heterozygosity, resulting in absent neurofibromin expression, is required for benign and malignant tumor formation (Bajenaru et al., 2001; Basu et al., 1992; Bollag et al., 1996; DeClue et al., 1992; Feldkamp et al., 1996; Guha et al., 1996; Gutmann et al., 1994, 2000; Lau et al., 2000; Legius et al., 1993; Powers et al., 2002; Side et al., 1997; Staser et al., 2013; Xu et al., 1992). In this respect, NF1-associated tumors from both genetically engineered mice and human surgical specimens exhibit markedly decreased or absent neurofibromin expression, increased RAS activity and elevated RAS-dependent effector pathway activation (Fig. 2B). As such, neurofibromin regulates RAS-MEK-ERK (also known as RAS-MAP2K-MAPK), RAS-AKT-mechanistic target of rapamycin (mTOR) and RAS-Rac1 signaling.

The most common tumors in individuals with NF1 are benign tumors involving peripheral nerve sheaths, called neurofibromas. These tumors are composed of neoplastic *NF1-*deficient Schwann lineage cells (Chen et al., 2019; DeClue et al., 1992; Kim et al., 1997; Le et al., 2011, 2009; Vogel et al., 1999; Zhu et al., 2002) and are classified as either cutaneous (discrete dermal) or plexiform (diffuse) neurofibromas. Although cutaneous neurofibromas are benign and do not progress to malignant tumors, 30-57% of NF1 patients will harbor a plexiform neurofibroma (pNF) (Brems et al., 2009; Mautner et al., 2008; Nguyen et al., 2011), which can undergo malignant transformation and progress to malignant peripheral nerve sheath tumors (MPNSTs). Heterozygous *NF1*-mutant (*NF1^+/−^*) and *NF1-*null (*NF1*^−/−^) Schwann cells from pNFs and MPNSTs exhibit reduced neurofibromin GAP activity and increased signaling downstream of RAS, including RAS-MEK-ERK and RAS-AKT-mTOR activation (Dombi et al., 2016; Endo et al., 2013; Gregorian et al., 2009; Gutmann et al., 1995c; Johannessen et al., 2005; Kawachi et al., 2013; Le et al., 2011, 2009).

Based on the finding of increased RAS activation in these tumors, farnesyltransferase inhibitors (FTIs), which inhibit post-translational modification (farnesylation) and membrane tethering of RAS proteins, were initially evaluated (Kim et al., 1997; Yan et al., 1995). Although *in vitro* treatments showed some ability to attenuate RAS pathway signaling, administration of FTIs *in vivo* was not successful in preventing pNF tumor progression in mice (Mahgoub et al., 1999) or in clinical trials with children with NF1 (Widemann et al., 2014a,b). Owing to increased mTOR complex signaling mediated by RAS in *NF1*-deficient mouse and human cells (Dasgupta et al., 2005b; Johannessen et al., 2005), along with early successes in preclinical mouse models of pNF and MPNST (Johannessen et al., 2008; Wu et al., 2012), phase 2 clinical trials with mTOR inhibitor, sirolimus, were initiated for pNF in children, which unfortunately had limited efficacy (Weiss et al., 2014). In recent years, there has been a greater focus on the use of MEK inhibitors for the treatment of nerve sheath tumors, based on promising preclinical studies (Jessen et al., 2013; Ramkissoon et al., 2019). Clinical trials using the MEK inhibitor, selumetinib, revealed decreased pNF and spinal neurofibroma burden (Jackson et al., 2020) with minimal side effects (Baldo et al., 2021), and another MEK inhibitor, trametinib, decreased tumor size (Toledano et al., 2021; Weiss et al., 2021). These exciting results culminated in MEK inhibitors, specifically selumetinib, becoming the first U.S. Food and Drug Administration (FDA)-approved agents for the treatment of progressive pNFs.

Grade 1 pilocytic astrocytomas of the optic pathway, also known as optic pathway gliomas (OPGs), are the second most common tumor in NF1, and are also driven by increased RAS-dependent signaling. In human surgical OPG biospecimens, *NF1* loss is associated with increased RAS and RAS effector activity (Lau et al., 2000), consistent with *Nf1* OPG mouse tumors, where loss of neurofibromin is associated with increased RAS signaling and RAS-ERK, RAS-AKT-mTOR activation (Dasgupta et al., 2005b; Kaul et al., 2015). Importantly, in this context, murine OPG formation reflects preferential activation of KRAS, rather than HRAS or NRAS (Dasgupta et al., 2005a), and operates in an mTOR-dependent manner (Banerjee et al., 2011). In addition, this RAS-dependent regulation of *Nf1* mouse optic glioma progression and growth is mediated by both MEK and PI3K/Akt activation, which can both converge to increase mTOR-dependent signaling (Banerjee et al., 2011; Kaul et al., 2015).

Although RAS inhibitors have not entered clinical trials for children with NF1-OPGs, both mTOR and MEK inhibitors have been tested as anti-tumoral therapies, based on promising studies in preclinical *Nf1*-OPG mouse models (Hegedus et al., 2008; Kaul et al., 2015). Despite the limited efficacy demonstrated for the mTOR inhibitor everolimus (Ullrich et al., 2021, 2020), a phase 2 selumetinib clinical trial in NF1 patients with grade 1 pilocytic astrocytomas reported significant tumor shrinkage (Fangusaro et al., 2019), supporting the notion that MEK inhibition may more effectively target neoplastic NF1-OPG tumor cells.

Additionally, individuals with NF1 occasionally develop pheochromocytomas and leukemias, most commonly myeloid leukemia and juvenile myelomonocytic leukemia (JMML), all of which are driven by aberrant RAS activity. As such, *NF1-*deficient pheochromocytomas, which are rare tumors developing from neural crest-derived chromaffin cells, exhibit RAS-mediated MEK/ERK1/2 hyperactivation (Park et al., 2005; Powers et al., 2002). Similarly, in leukemias, *NF1* loss in patient (Side et al., 1997) or murine (Bollag et al., 1996; Hiatt et al., 2001; Zhang et al., 1998) hematopoietic precursors, results in increased RAS-MEK/ERK pathway activation (Kim et al., 2007; Zhang et al., 1998). Although FTIs have little efficacy against disease progression in preclinical models (Mahgoub et al., 1999), MEK1/2 inhibition in a JMML murine model reduced hematopoietic progenitor proliferation *in vitro* and myelopoiesis *in vivo* (Staser et al., 2013). As MEK inhibitors are emerging as promising agents for the treatment of NF1-associated tumors, current studies aim to define the tolerance profiles in children and the design of therapeutic regimes that reduce drug resistance.

### Tissue-specific RAS signaling

RAS hyperactivation is observed in human and mouse NF1-associated tumors, and similar increases are also observed in non-neoplastic cells in a tissue-specific manner. In this regard, reduced *Nf1* expression in central nervous system neurons also results in increased RAS activation and impaired neurite extension, while in peripheral nervous system neurons, reduced *Nf1* expression results in increased RAS/Akt-mediated neurite extension (Brown et al., 2012, 2010b).

In addition, differential engagement of downstream RAS signaling molecules has been described (Fig. 2B). For example, loss of neurofibromin expression in mouse neural stem cells results in increased RAS activation, operating through different RAS effectors that either govern cell growth or cell differentiation (Chen et al., 2015). As such, neurofibromin-RAS-PI3K-Akt signaling controls neural stem cell proliferation, whereas multi-lineage differentiation is regulated by RAS-MEK signaling. Moreover, mTOR activation and increased neural stem cell growth are also dictated by the expression of the mTOR-associated protein, RICTOR. Analysis of RICTOR expression reveals tremendous variability in different populations of brain ventricular neural stem cells, which has differential effects on their proliferation (Lee et al., 2010). Finally, the cell-type-specific expression of other mTOR complex components, like the G-protein-coupled receptor kinase-interacting protein-1 (GIT1), also create unique mTOR signaling complexes important for the survival of brain cells (astrocytes), but not non-neural cell types (Smithson and Gutmann, 2016).

Additionally, some cell types, like microglia, exhibit increased RAS-dependent JNK activation (Fig. 2B), such that JNK inhibition by SP600125 inhibits microglia activation *in vitro* and reduces *Nf1* OPG proliferation *in vivo* (Daginakatte et al., 2008). Similarly, RAS-Tiam-Rac1 hyperactivation in *Nf1*-deficient cells (Fig. 2B) has been shown to underlie pNF formation in mice (Mund et al., 2020). Finally, RAS-Rac1-PAK signaling (Fig. 2B) in neurons governs social learning in *Nf1*^+/−^ mice (Molosh et al., 2014), while, in *Nf1*^+/−^ mast cells, it regulates gain-of-function phenotypes relevant to neurofibroma pathogenesis (McDaniel et al., 2008)

## Neurofibromin and non-canonical RAS pathway signaling

While complete loss of *NF1* expression is necessary for tumor formation, heterozygosity for a germline mutation can cause significant cognitive and behavioral impairments in children with NF1. As such, more than 70% of children with NF1 will manifest attention, learning, language and executive function impairments (Eby et al., 2019; Hyman et al., 2005; Ozonoff, 1999; Soucy et al., 2012). Despite these common and quality-of-life-affecting comorbidities, the precise molecular pathways underlying these clinical features remain incompletely understood. Preclinical invertebrate (*Drosophila melanogaster*) and vertebrate (mouse, minipig) models of NF1 also exhibit behavioral defects, and have provided insights into the neurofibromin-regulated brain circuitry that governs cognitive function.

### Neurofibromin GABA regulation

In *Nf1* murine models, heterozygosity for a germline *Nf1* mutation (*Nf1*^+/−^ mice) is sufficient to cause social behavioral, spatial learning and hippocampal long-term potentiation (LTP) deficits (Costa et al., 2002; Cui et al., 2008; Molosh et al., 2014; Silva et al., 1997). These *Nf1^+/−^* mice exhibit increased inhibitory postsynaptic potentials in the prefrontal cortex and striatum, as well as defects in LTP, both of which result from increased GABAergic neuron excitation and signaling (Costa et al., 2002; Cui et al., 2008; Shilyansky et al., 2010). Similarly, human neural progenitor cell (NPC)-derived GABAergic neurons harboring different *NF1* mutations found in NF1 patients all exhibit increased GABA levels, irrespective of the underlying germline *NF1* gene mutation (Anastasaki et al., 2020).

Although the exact mechanism underlying neurofibromin control of GABA levels has yet to be elucidated, it is known that decreased neurofibromin expression causes RAS hyperactivation in *NF1*^+/−^ neurons, which in turn results in increased GABA transmission. Conversely, genetic depletion of KRAS or NRAS in *Nf1*^+/−^ mice (*Nf1^+/−^;KRAS^+/−^* and *Nf1^+/−^;NRAS*) normalizes the RAS hyperactivation and reverses the cognitive deficits (Costa et al., 2002; Cui et al., 2008). In addition, non-specific inhibition of RAS farnesylation and geranylgeranylation by lovastatin corrects the spatial learning and memory deficits in these mice (Li et al., 2005). Unfortunately, clinical trials using lovastatin and simvastatin failed to improve cognitive and behavioral deficits in children with NF1 (Bearden et al., 2016; Krab et al., 2008; Payne et al., 2016; van der Vaart et al., 2013). Because these farnesyltransferase inhibitors also have RAS-independent effects, additional studies using MEK inhibitors have been undertaken and reported attenuated ERK signaling and improved learning in *Nf1*^+/−^ mice (Cui et al., 2008). Moreover, GABA_A_ receptor inhibition with picrotoxin (Costa et al., 2002; Omrani et al., 2015) rescues LTP deficits in *Nf1*^+/−^ mice, irrespective of RAS hyperactivation, suggesting that RAS activity alone may not be sufficient to regulate GABA-regulated LTP and mouse learning. Collectively, these studies reveal that some NF1-associated behavioral deficits are attributed to increased GABA transmission, and neurofibromin control of neuronal GABA is not solely regulated by RAS signaling.

### Neurofibromin cyclic AMP regulation

Pioneering work in *Drosophila* revealed that loss of the *NF1* ortholog (*dNf1*; also known as *Nf1*) causes reductions in body size (Walker et al., 2013), as well as defects in associative learning (Guo et al., 2000), short-term memory (Guo et al., 2000; King et al., 2016), circadian rhythm (Bai et al., 2018; Machado Almeida et al., 2021) and grooming behaviors (King et al., 2016). However, these specific deficits were not directly attributed to RAS deregulation but were caused by neurofibromin regulation of cyclic AMP (cAMP)/PKA signaling. Specifically, reduced potassium currents arise in *dNf1* mutants due to diminished activation of adenylyl cyclase and cAMP production, which are rescued by pharmacologic restoration of cAMP levels (Guo et al., 1997). Likewise, expression of the catalytic subunit of cAMP-dependent PKA, or overexpression of a human *NF1* transgene, ameliorates the cAMP-driven small body size and adenylyl cyclase activity defects in *dNf1* flies (Buchberg et al., 1990; Tong et al., 2002).

While cAMP was necessary to restore *dNf1-*mutant fly deficits, reintroduction of the neurofibromin GAP domain in these mutants restored cAMP levels, suggesting that neurofibromin-RAS signaling regulates cAMP homeostasis. As such, introduction of the GAP domain restored cAMP-dependent long-term memory and body size deficits in *dNf1*-mutant flies (Walker et al., 2006). In striking contrast, the control of short-term memory in flies is RAS independent and regulated by the C-terminal domain of neurofibromin (Ho et al., 2007). Mirroring *Nf1-*mutant mouse findings, *dNf1* loss results in increased GABAergic signaling and disrupted associative learning. Importantly, learning is not restored following pharmacologic cAMP restoration, indicating that GABAergic signaling occurs independently of cAMP/PKA signaling in *dNf1*-mutant flies (Georganta et al., 2021). Together, these observations reveal RAS-cAMP-dependent long-term memory and RAS-cAMP-independent short-term memory functions of neurofibromin in *Drosophila* neurons.

**Fig. 3.**
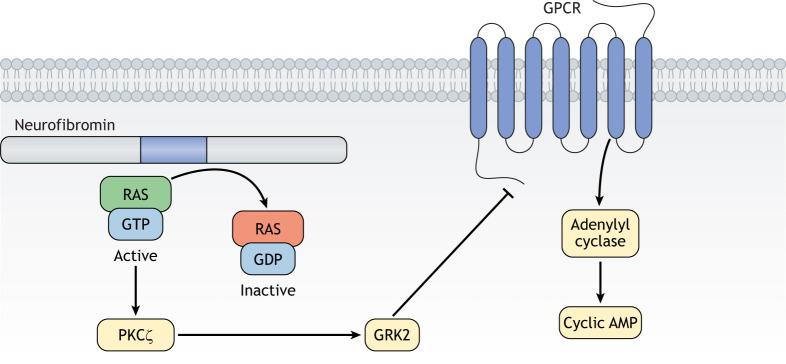
**Non-canonical RAS signaling.** Neurofibromin controls cyclic AMP levels by regulating the PKCζ molecule to control GRK2 activity, leading to inhibition of GPCR signaling and reduced adenylyl cyclase-mediated generation of cyclic AMP. GPCR, G-protein-coupled receptor; PKCζ, protein kinase C-zeta.

Analogous to observations made in flies, neurofibromin reduction in mouse *Nf1^+/−^* hippocampal neurons and in human *NF1^+/−^* NPCs results in reduced cAMP levels, shorter axonal lengths and smaller growth cone areas (Anastasaki and Gutmann, 2014), which are restored after pharmacologic cAMP elevation (Brown et al., 2010b). Importantly, the same cAMP-driven neurite deficits are rescued by genetic or pharmacologic reduction of RAS activity (Anastasaki and Gutmann, 2014), but not MEK or PI3K inhibition (Brown et al., 2010b). Combined with the results from *Drosophila* models, these findings suggest that cAMP homeostasis is dependent upon neurofibromin control of RAS signaling, but that RAS-driven cAMP regulation is MEK/AKT/mTOR independent. Using a combination of human induced pluripotent stem cell (hiPSC)-derived neurons and mouse primary neuron cultures, we found that neurofibromin regulates neuronal cAMP homeostasis through non-canonical RAS-mediated phosphorylation of PKCζ (also known as PRKCZ) and subsequent GRK2-dependent desensitization of GPCR activation of adenylyl cyclase (Anastasaki and Gutmann, 2014) (Fig. 3). As such, axonal length defects in *Nf1*^+/−^ murine hippocampal neurons and human NPCs are restored following genetic or pharmacologic blockade of PKCζ.

## RAS-independent neurofibromin function

Given that neurofibromin is a large molecule with numerous protein-binding domains and novel conformational states (Naschberger et al., 2021), it is likely that other, perhaps RAS-independent, properties exist.

### Neurofibromin regulation of dopamine homeostasis

*NF1* mutation in both human and mouse neurons leads to reduced levels of the neurotransmitter, dopamine (Anastasaki et al., 2015; Brown et al., 2010a; Diggs-Andrews et al., 2013), and pharmacological elevation of dopamine (L-DOPA) reverses spatial learning and memory abnormalities exhibited by heterozygous *Nf1*-mutant mice (Diggs-Andrews et al., 2013). We also discovered that different *NF1* germline mutations in mouse and human neurons, NPCs and 3D cerebral organoids result in different levels of neurofibromin and dopamine expression, ranging from <25% to >75% reductions relative to control cells (Anastasaki et al., 2020, 2015). Importantly, regardless of neurofibromin expression levels, all heterozygous *NF1*-mutant neurons have similar levels of RAS hyperactivation. In contrast, dopamine levels closely parallel neurofibromin expression, suggesting that neurofibromin regulation of dopamine production operates through RAS-independent mechanisms. Given the interactions between inhibitory GABAergic and excitatory dopaminergic neurons in the brain regions that are important for learning and memory, it is pertinent to consider the possibility that RAS-GABA signaling in inhibitory neurons could indirectly modulate dopamine homeostasis.

### Neurofibromin regulation of HCN channel function

A signature property of neurons is their ability to elicit action potentials causing neuron excitation. In addition to exhibiting defects in neuronal morphology (neurite lengths), brain and peripheral sensory neurons from heterozygous *Nf1-*mutant mice are hyperexcitable at baseline relative to their wild-type counterparts. As such, peripheral sensory neurons from adult *Nf1*^+/−^ mice exhibit increased numbers of action potentials in response to depolarizing current, as well as lower firing thresholds, lower rheobase currents and shorter firing latencies (Wang et al., 2005). In the central nervous system, light-induced stimulation of *Nf1-*mutant, but not wild-type, retinal ganglion cell neurons increased OPG growth in mice, while light deprivation halted tumor formation in OPG-prone *Nf1-*mutant mice (Pan et al., 2021). These findings imply that *Nf1-*mutant neurons are uniquely sensitive to modulation of excitation and electrical activity.

Although the mechanisms underlying hyperexcitability in *Nf1*-mutant neurons have not been fully elucidated, both *Nf1^+/−^* mice and mice harboring a neuron-specific inactivating *Nf1* mutation (*Nf1^9a−/9a−^*) exhibit increased GABAergic interneuron excitability (Omrani et al., 2015). This elevated excitability results from attenuated activity of hyperpolarization-activated cyclic nucleotide-gated channel 1 (HCN1), a protein that binds to the N-terminal domain of neurofibromin (Omrani et al., 2015) and modulates neuronal inward cationic currents (I_h_) (Wahl-Schott and Biel, 2009). Restoration of HCN1 channel activity following treatment with a HCN channel agonist, lamotrigine, reverses the spatial learning deficits in *Nf1*-mutant mice. Importantly, neurofibromin HCN channel-regulated I_h_ influx is completely independent of RAS activity, because constitutively active HRAS (*H-RAS^G12V^*)-expressing mice have I_h_ levels similar to those of wild-type mice (Omrani et al., 2015). Together, these data suggest that neurofibromin-dependent HCN1 channel activity controls neuronal excitability in a RAS-independent manner.

### Neurofibromin regulation of estrogen receptor activity and cell cycle progression

*NF1* mutation or loss are common genetic events both in sporadic (Stephens et al., 2012) and NF1-associated (Uusitalo et al., 2016) breast cancer, where *NF1* loss was associated with increased tumor aggressiveness and poor patient prognosis. Population studies on breast cancer samples revealed a correlation between *NF1* loss and upregulation of estrogen receptor (ER)-associated pathways in human breast cancer tissues, as well as increased ER activity in rat models of mammary adenocarcinoma (Dischinger et al., 2018). Building upon this novel relationship, neurofibromin can translocate to the nucleus of ER^+^ breast cancer cell lines to bind ER and act as its transcriptional co-repressor (Zheng et al., 2020). As such, loss of *NF1* expression in these cell lines leads to global enhancement of ER-driven transcription and tamoxifen agonism of ER, such that tamoxifen administration promotes cell growth and, conversely, estradiol hypersensitivity. Importantly, the binding of neurofibromin to the ER and its ability to function as an ER repressor are independent of RAS signaling, as neither RAF nor MEK inhibition attenuated estrogen receptor transcription. Additionally, independent of RAS-GAP function, neurofibromin regulates the metaphase-to-anaphase transition. In this respect, the C-terminal domain of neurofibromin associates with the mitotic spindles to induce spindle damage and mitotic arrest (Koliou et al., 2016; Luo et al., 2014). Collectively, these studies identify a novel binding partner and highlight the ability for neurofibromin to regulate cell proliferation in a non-RAS-mediated fashion.

## Neurofibromin isoforms and subcellular localization

Although there is a wealth of information delineating the varied functions of neurofibromin in different cell types and tissues, most of these studies are focused on RAS pathway regulation. The recent elucidation of the physical structure neurofibromin revealed that it likely functions as a homodimer (Lupton et al., 2021; Naschberger et al., 2021; Sherekar et al., 2020) with at least two distinct molecular conformations. In its closed, auto-inhibited conformation, RAS binding is blocked, thus inhibiting neurofibromin RAS-GAP activity. However, in its open conformation, the lipid-binding SEC-PH domain of one neurofibromin protomer interacts with the cell membrane to expose and activate the RAS-GAP domain (Naschberger et al., 2021). Neurofibromin forms dimers to bind and hydrolyze RAS molecules, and neurofibromin monomers and dimers are similarly efficient in their RAS-GAP activity *in vitro* (Sherekar et al., 2020). As structural studies evolve, it will become important to define the bioavailability of neurofibromin as a monomer or a dimer *in vivo*, which could partly account for cell- or tissue-specific differences in neurofibromin binding and downstream signaling pathway transduction.

**Fig. 4.**
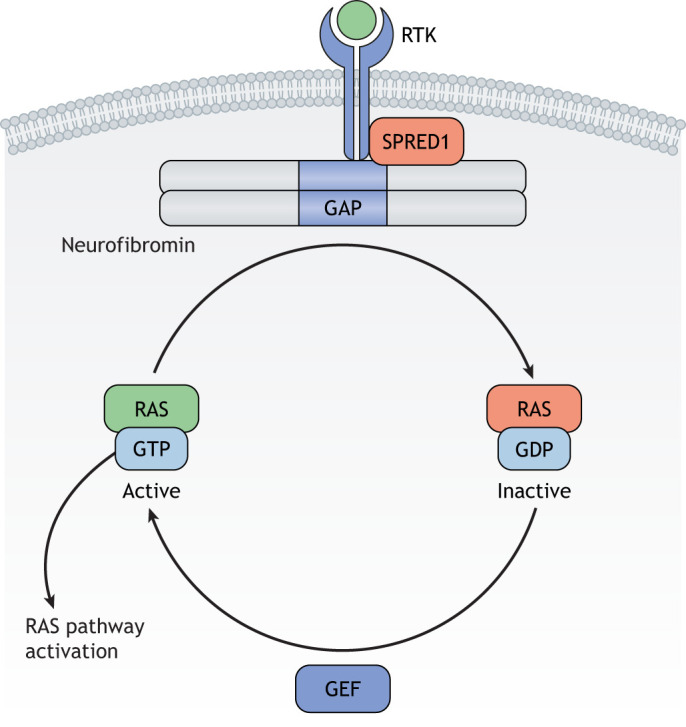
**Neurofibromin subcellular localization.** Upon mitogen stimulation and subsequent RTK activation, SPRED1 recruits neurofibromin to the plasma membrane. Neurofibromin, in turn, acts as a dimer to hydrolyze the conversion of active RAS-GTP to inactive RAS-GDP. RTK, receptor tyrosine kinase.

Neurofibromin requires SPRED1 to localize to the plasma membrane (Dunzendorfer-Matt et al., 2016; Stowe et al., 2012), but it is predominately expressed in the cytoplasm. However, it is conceivable that binding partners other than SPRED1 alter its subcellular localization and/or facilitate interactions with proteins that operate independently of RAS signaling (Fig. 4). In this respect, neurofibromin has previously been reported within the nucleus of neurons and glia (Daston et al., 1992; Koliou et al., 2016; Li et al., 2001), as well as in the nucleus of breast cancer cells (Dischinger et al., 2018; Zheng et al., 2020), as a result of interactions with Ran and CRM-1 (also known as XPO1) (Zheng et al., 2020).

Moreover, at least four alternatively spliced neurofibromin isoforms have been reported. The first one includes exon 30alt31 (formerly exon23a), which is located within the RAS-GAP domain. The levels of this isoform vary depending on the developmental stage examined, but it tends to be more highly expressed in differentiated cells (Gutmann et al., 1995b). Exon 30alt31 inclusion results in reduced RAS-GAP activity, and its expression causes behavioral deficits in mice (Costa et al., 2001; Nguyen et al., 2017). The alternatively spliced exon 11alt12 (formerly exon 9a) is located within the amino terminus of the protein, in a locus lacking obvious structural domains. This isoform is intriguing, as its expression is exclusively restricted to postnatal brain neurons (Danglot et al., 1995; Geist and Gutmann, 1996; Gutmann et al., 1999), and, as such, might mediate other central nervous system-specific functions of neurofibromin. Additionally, isoform exon 56alt57 (formerly 48a) is exclusively found in heart and muscle tissue (Gutmann et al., 1995a), whereas isoform exon 12alt13 (formerly 10a-2) is ubiquitously concentrated in perinuclear granular structures, supporting a non-plasma membrane function for neurofibromin (Kaufmann et al., 2002). Finally, an isoform with a 140 bp deletion in exon 21 was recently identified exclusively in a breast cancer cell line, where it might promote tumor progression (Dischinger et al., 2018). Taken together, these findings highlight the diversity of the neurofibromin protein and underscore the importance of tissue- and cell-specific analyses of its function.

## Conclusions

As we learn more about neurofibromin binding partners, tissue-specific functions and RAS-independent regulatory properties, it is likely that more precise therapeutic agents will emerge to selectively and effectively treat some of the clinical manifestations of NF1 in patients. To date, although targeted MEK inhibition has been the most effective avenue in eliciting tumor reduction responses, the success is not universal for all NF1-associated tumors, and the long-term efficacy of this treatment remains unknown. Moreover, as the majority of patients with NF1 develop non-tumor-related comorbidities (e.g. cognitive deficits), a subset of which are not dependent on RAS-MEK activity, the identification of RAS-independent targetable proteins or pathways will be critical for their management. Finally, using NF1 as a platform to uncover cell- and tissue-specific RAS-dependent signaling pathways is likely to improve our understanding of the molecular pathology associated with other RASopathies or other RAS-driven cancers.
